# Contributions to cytogenetics of *Plectranthus barbatus* Andr. (Lamiaceae): a medicinal plant

**DOI:** 10.3897/CompCytogen.v9i3.5164

**Published:** 2015-07-24

**Authors:** Aryane Campos Reis, Lyderson Facio Viccini, Saulo Marçal de Sousa

**Affiliations:** 1 Universidade Federal de Juiz de Fora, Departamento de Biologia/Laboratório de Genética 36036-900, Juiz de Fora, MG, Brazil

**Keywords:** AT/GC content, chromosome number, heterochromatin, genome size, molecular cytogenetics

## Abstract

Accessions of *Plectranthus barbatus* (Lamiaceae), a medicinal plant, were investigated using a cytogenetic approach and flow cytometry (FCM). Here, we describe for the first time details of the karyotype including chromosome morphology, physical mapping of GC rich bands (CMA_3 _banding), as well as the mapping of 45S and 5S rDNA sites. All accessions studied showed karyotypes with 2*n* = 30 small metacentric and submetacentric chromosomes. The CMA_3_ banding and fluorescent *in situ* hybridization techniques revealed coincidence between CMA_3_ bands and 45S rDNA sites (6 terminal marks) while for the 5S rDNA were observed 4 subterminal marks no coincident with CMA_3 _marks. For nuclear genome size measurement, the FCM procedure provided histograms with G_0_/G_1 _peaks exhibiting CV between 2.0–4.9 and the mean values obtained for the species was 2C = 2.78 pg, with AT% = 61.08 and GC% = 38.92. The cytogenetic data obtained here present new and important information which enables the characterization of *Plectranthus barbatus*.

## Introduction

The genus *Plectranthus* L' Herit. (Lamiaceae) contains nearly 300 species distributed in tropical Africa, Asia, Australia and Brazil (Lukhoba et al. 2006, Alasbahi and Melzigh 2010). Many species show important medicinal properties and a large variation in morphology, chemistry as well as in the chromosome number, ranging from 2n = 14 to 2n = 84 (Morton 1962, De Wet 1958, Lukhoba et al. 2006, Alasbahi and Melzig 2010).

One of the most important species traditionally used in folk medicine, *Plectranthus barbatus* Andr., shows a large morphological variation and is also commonly cited by innumerous synonyms such as *Plectranthus forskohlii* Briq, *Plectranthus forskalaei* Willd., *Plectranthus kilimandschari* (Gürke) H. L. Maass., *Plectranthus grandis* (Cramer) R. H. Willemse, *Coleus forskohlii* Briq., *Coleus kilimandschari* Gürke ex Engl., *Coleus coerulescens* Gürke, *Coleus comosus* A. Rich., and *Coleus barbatus* (Andr.) Benth (Lukhoba et al. 2006, Alasbahi and Melzig 2010).

Popularly, *Plectranthus barbatus* is mainly used for liver disturbance, respiratory disorders, heart diseases and certain central nervous system disorders, being also used as hypotensive and antispasmodic (Alasbahi and Melzig 2010). Due to these broad applications, numerous chemical and pharmacological studies have been done showing forskolin also called coleonol as the major active component (Lukhoba et al. 2006, Alasbahi and Melzig 2010).

In spite of intensive pharmacological studies, few studies have been done about biological aspects of the species. Considering the medicinal importance of *Plectranthus barbatus* and a large number of synonyms reported, basic information such as karyotypic traits are very important, helping the correct plant identification and also the production of commercial varieties in breeding programs (Sousa et al. 2009, Ferreira et al. 2010, Sousa et al. 2010, Pierre et al. 2011, Sousa et al. 2012, Sousa et al. 2013, Reis et al. 2014, Viccini et al. 2014). Regarding cytogenetic studies, only the chromosome number and meiosis behavior were reported so far (De Wet 1958, Morton 1962). Detailed data such as chromosome banding and molecular cytogenetic traits are not available for *Plectranthus* species.

The aim of the present work was to describe new chromosome markers for *Plectranthus barbatus*, by using chromosome banding and molecular cytogenetic techniques. The genome size and AT/GC content by flow cytometry were also reported to help the characterization of different cytotypes of *Plectranthus barbatus* as well as to understand the taxonomy and evolution of the genus *Plectranthus*.

## Material and methods

### Plant material

Five accessions of *Plectranthus barbatus* were collected at Juiz de Fora, Latitude: 21°45'51"S and Longitude: 43°21'01"W, Minas Gerais, Southeast Brazil and cultivated in a greenhouse of Federal University of Juiz de Fora. The herbarium voucher specimens of each accession were deposited at the CESJ Herbarium of Federal University of Juiz de Fora with following numbers: PB 2324, PB 2325, PB 2326, PB 2327 and PB 2328.

### Preparation of mitotic spreads

Roots tips were pre-treated with 8-hydroxyquinoline solution (0.003 M) at room temperature for 7h and then fixed in ethanol and acetic acid (3:1 v/v) for 24h at -20 °C. Root meristems were submitted to enzymatic maceration (4% Celullase: 40% Pectinase) for 5h at 37 °C. The slides was prepared according to Carvalho and Saraiva (1993, 1997).

### Determination of morphological chromosome parameters

Chromosome length, short and long arms and ratio between chromosome arms (AR) were measured on 5 well-spread metaphases for each accession using the CellSens software (Olympus, Tokyo, Japan). Chromosome classification was done according to Levan et al. (1964). The ideogram were drawn based on centromeric index and arranged in the decreasing size order.

### Molecular cytogenetics

Fluorescence *in situ* hybridization (FISH) was performed using the probe pTA71 from *Triticum aestivum*, which contain a 9kb EcoRI fragment including the 18S – 5.8S – 25S rRNA gene and intergenic spacer regions (rDNA) (Gerlach and Bedbrook 1979) and 5S probes from *Zea mays* (D.-H. Koo and J. Jiang, University of Wisconsin, unpublished data), kindly provided by Dr. J. Jiang. Each probe was labeled with digoxigenin by nick translation and then hybridized according to Jiang et al. (1995) with minor modifications. The hybridization mixture was denatured at 85 °C for 10 min and immediately transferred to an icebox. The slides were denatured at 85 °C for 1 min and treated with a series of alcohol washes (70%, 90%, and 100% ethanol for 5 min each). The hybridization mixture was then added to the slides and the chromosomes allowed to hybridize at 37 °C for 48 h in a humidified chamber. Posthybridization washes were carried out using 2 × SSC buffer (0.3 mol/L sodium citrate, 0.03 mol/L sodium chloride, pH 7) and 1 × PBS buffer (0.136 mol/L sodium chloride, 0.27 mol/L potassium chloride, 0.1 mol/L dibasic sodium phosphate, 0.2 mol/L monobasic potassium phosphate, pH 7.4). Probes were detected with anti-DIG conjugate with rhodamine (Sigma) and postdetection washes were performed using 1 × TNT buffer (0.1 mol/L Tris, 0.15 mol/L sodium chloride, 0.05% Tween-20) and 1 × PBS at room temperature. Chromosomes were counterstained with 2 µg/mL of DAPI (Sigma). The slides were mounted in Vectashield (Vector, Burlingame, California, USA), and some samples were rehybridized after discoloration in 100% ethanol for 24h. Good metaphases were captured in an Olympus DP72 digital camera and images with DAPI, 45S and 5S signals were merged using CellSens software (Olympus, Tokyo, Japan). Chromosomes were observed using an epifluorescence microscope (Olympus BX 51) with appropriate filter set (Olympus, Tokyo, Japan).

### Chromosome banding

The chromosome banding was performed according Schweizer (1976). Aged slides were stained with chromomycin A_3 _(0.5 mg/mL) for 1 h, dystamicyn (0.1 mg/mL) for 30 min and 2-4 diamidino-2-phenylindole (2 µg/mL) for 30 min. The slides were mounted in Mcllvaine’s pH 7.0 buffer-glycerol (1:1 v/v). For this analysis five metaphases of each accession were observed and captured in an Olympus DP72 digital camera. The chromosomes were observed using an epifluorescence microscope (Olympus BX 51) with appropriate filter set.

### Flow Cytometry (FCM)

Nuclear DNA content was determined according to the method of Galbraith et al. (1983). Approximately 20–30 mg of young and fresh leaves for each accessions of *Plectranthus barbatus* and the same amount of young foliar tissue of standard references *Zea mays* CE-777 were chopped on ice with 1 mL of OTTO I lysis buffer solution (Otto 1990) supplemented with 50 µg mL^–1^RNAse. The suspension was filtered through 40nm mesh into 2 mL microcentrifuge tube and centrifuged at 1,100 rpm for 5 minutes. The pellet was incubated in 100 µL OTTO I lysis buffer for 10 minutes and then was added 1.4 mL of OTTO I: OTTO II (1:2 v/v) buffers. The sample were homogenized and stained with 50 µg mL^–1^of propidium iodide (PI) to determine the total DNA content. AT/GC composition was determined by adding DAPI (4,6-diamidino-2-phenylindole) 4 µg mL^–1^ to the samples. At least 10,000 nuclei were analyzed per sample in a FACSCantoII (BectonDickinson) flow cytometer. The histograms were analyzed using Flowing 2.5.1 software (http://www.flowingsoftware.com).

The DNA nuclear amount (pg) of each sample was estimated by the relative fluorescence intensity of the sample and the internal reference standard (*Zea mays* 5.43). Each accession was measured three times following the equation (Dolezel 2003):


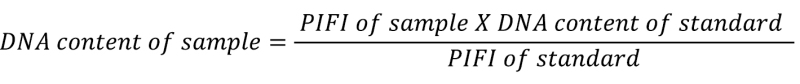


where PIFI is the fluorescence intensity of cells stained with propidium iodide in G1 stage.

The AT percentage of P. barbatus was measured in relation to Zea mays reference standard, following the equation described by Godelle et al. (1993):


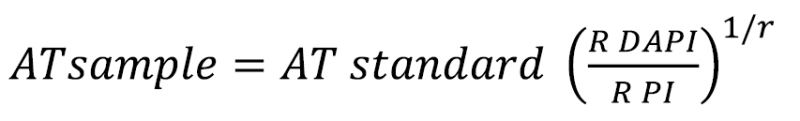


where R is the ratio of fluorescence intensity between the peak of P. barbatus and Zea mays, and r (binding length) = 3 for DAPI dye (Meister and Barow 2007). The percentage of the complementary bases was calculated as GC%= 100 – AT%.

## Results

The accessions showed symmetrical karyotype, all with 2n = 30. Fourteen chromosomes showed centromeres at the median (m, AR = 1–1.7) and one of them at submedian region (sm, AR = 1.71–3.0) (Table 1). No secondary constrictions were observed. Chromosome lengths ranged from 2.51–1.86 µm (Table 1) and the Karyotype formulae (KF) was KF = 14m+1sm.

Relative chromosome length revealed that the larger chromosome represented around 7.91% of the genome size and the shortest one 5.86% (Table 1).

**Table 1.** Chromosome morphometry of *Plectranthus barbatus* and estimative of DNA content for each chromosome.

Chromosome	Relative lenght (%)	Absolute lenght (µm)	Short arm length (µm)	Long arm length (µm)	Arm ratio	Classification	DNA pg/chromosome	Mpb/chromosome
1	7.91	2.512	1.127	1.385	1.22	m	0.109	107.453
2	7.45	2.362	0.995	1.367	1.37	m	0.103	101.204
3	7.07	2.247	0.900	1.347	1.49	m	0.098	96.042
4	7.05	2.242	1.050	1.192	1.13	m	0.097	95.770
5	6.97	2.220	0.885	1.335	1.50	m	0.096	94.683
6	6.68	2.120	0.980	1.140	1.16	m	0.092	90.744
7	6.65	2.112	0.995	1.117	1.12	m	0.092	90.336
8	6.55	2.080	0.940	1.140	1.21	m	0.090	88.978
9	6.46	2.047	0.815	1.232	1.51	m	0.089	87.755
10	6.43	2.047	0.842	1.205	1.43	m	0.089	87.348
11	6.41	2.040	0.837	1.202	1.43	m	0.089	87.076
12	6.25	1.980	0.825	1.155	1.40	m	0.086	84.903
13	6.18	1.960	0.702	1.257	1.79	sm	0.085	83.952
14	5.98	1.895	0.812	1.082	1.33	m	0.083	81.235
15	5.86	1.865	0.857	1.007	1.17	m	0.081	79.605

**Figure 1.**
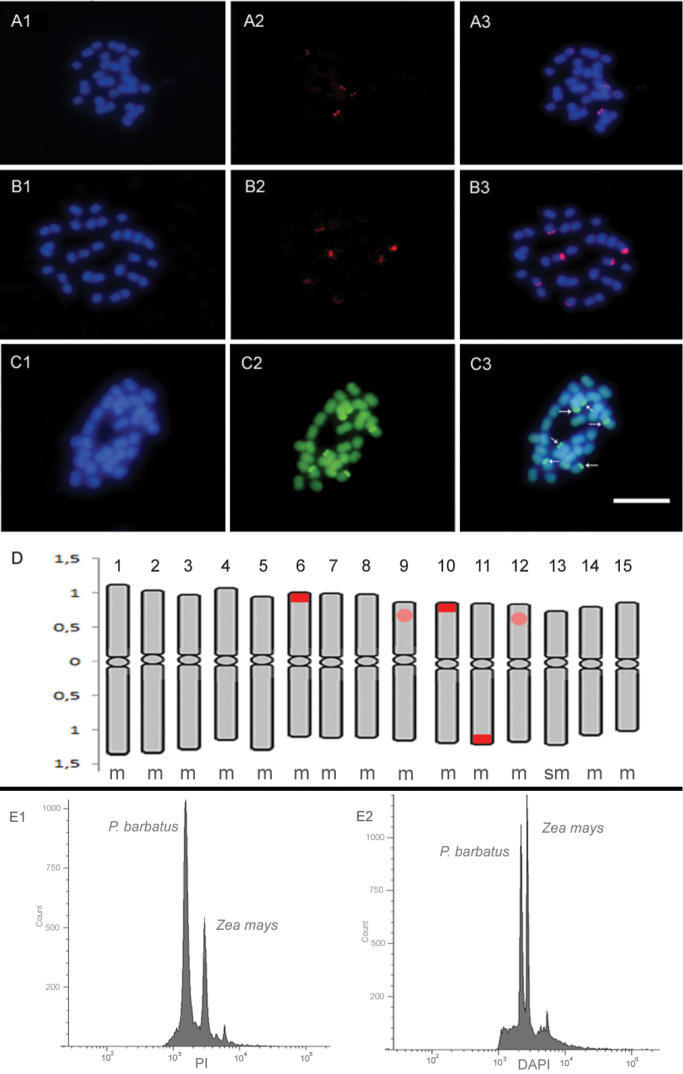
Representative metaphases of 5S rDNA (**A1, A2, A3**) and 45S rDNA (**B1, B2, B3**), DAPI/CMA_3_ banding (**C1, C2, C3**), Ideogram of *Plectranthus barbatus* (**D**) (light red circle = 5S rDNA, dark red rectangle = 45S rDNA), Flow cytometry histograms (**E1** = propidium iodide - DNA total amount, **E2** = DAPI - AT content). Bar = 5 µm.

The 45S rDNA signal were observed in three chromosome pairs on the terminal portion (two in the short arms of chromosomes 6 and 10, and one in the long arm of chromosome 11) (Fig. 1 B1–B3), while 5S rDNA signals were observed in subterminal portion of two chromosome pairs, in the short arm of chromosomes 9 and 12, respectively (Fig. 1 A1–A3). The 45S rDNA sites showed greater bands when compared with those ones observed for 5S rDNA sites, which showed pairs of little dots (Fig. 1 A2–A3 and B2–B3).

No centromeric, interstitial or terminal DAPI bands were observed. However, fluorochrome staining with CMA_3 _revealed bands on three chromosome pairs, which were also DAPI negative. Heterochromatin blocks correspond to 0.37% of the total haploid complement. The observed bands showed similar size and bright, all of them at terminal regions and coincident with 45S rDNA marks, on the short arm of chromosome 6 and 10, respectively, and on the long arm of chromosome 11 (Fig. 1C, D). No additional CMA bands were observed in any of the chromosome pairs.

Regarding to the nuclear genome size estimation, the flow cytometry (FCM) technique provided high quality histograms with G_0_/G_1 _peaks showing CV = 2,0–4.9. The 2C DNA content estimated for the species was 2C = 2.78 pg (Table 1). Taking into account that 1pg = 978 Mpb (Dolezel et al. 2003), and combining cytometric and cytogenetic data the largest chromosome (chromosome 1) corresponds to 107.452 Mpb (~7% of the genome), while the smallest one (chromosome 15) corresponds to 79.605 Mpb (~6% of the genome). By using FCM *Plectranthus barbatus* genome (2C) possess 2714.148 Mpb (Table 1).

In addition, PI and DAPI fluorochromes index allowed, for the first time, the estimation of base composition of the genome of *Plectranthus barbatus*. The percentage of base was 61.08% for AT and 38.92% for GC. The representative histograms of DNA content and base composition can be seen in Figure 1E.

## Discussion

The genus *Plechtrantus* has a great variability of chromosome numbers. Although the basic chromosome number for most of the species is x = 7, and 2n chromosome number is 28, some species show secondary basic chromosome numbers (6 and 8) (De Wet 1958, Morton 1962). Additionally, intraspecific chromosome numbers variations are common in the genus. Morton (1993) described, for example, the following aneuploid numbers: 2n = 26 and 28 for *Plechtrantus assurgens* (Baker) J. K. Morton and *Plechtrantus glandulosus* Hook, 2n = 28 and 30 for *Plechtrantus tenuicaulis* (Hook. f.) J. K. Morton, while for *Plechtrantus amboinicus* (Lour.) Spreng, Thoppil (1993) described a large variation of somatic numbers (2n = 16, 24, 30, 32, 34 and 48), indicating that polyploidy events, in association with aneuploidy might contributed to the genus complexity.

Many authors suggested that *Plectranthus barbatus* can be cytologicaly considered as a species with different cytotypes that include a possible aneuploid series with 2n = 28, 30, 32 and 34 described so far (Reddy 1952, Riley and Hoff 1961, Saggoo and Bir 1983). In our study all individuals showed 2n = 30, corroborating one of the numbers previously described (Cherian and Kuriachan 1981, Saggoo and Bir 1983, Bahl and Tyagi 1988, Thoppil 1993). If we consider x = 7 as the main basic chromosome number for the genus *Plechtrantus*, as was suggested by most of the authors, and the same number as the basic chromosome number for *Plectranthus barbatus*, 2n=30 could be a consequence of both polyploidy and aneuploidy events however meiotic behaviour studies are necessary to understood this condition.

Chromosome length and chromosome classification of *Plectranthus barbatus* here observed is very similar to those ones already described for other cytotypes of *Plectranthus barbatus* (De Wet 1958, Morton 1962, Cherian and Kuriachan 1981, Saggoo and Bir 1983, Bahl and Tyagi 1988, Thoppil 1993). Nevertheless, some karyotypic formulae described are different from our data indicating that the genome of the species, apart from chromosome number variation, is also very instable regarding the chromosome structure (De Wet 1958, Morton 1962, Bahl and Tyagi 1988, Thoppil 1993). Chromosome morphological alterations such as deletions, duplications, inversions, transpositions and translocations may have occurred independently over time in different accessions of *Plectranthus barbatus*. These rearrangements are very common in polyploids due to the genetic redundancy observed after the genome duplication, event known as “genome shock” (Lim et al. 2008, Reis et al. 2014).The individuals with extra copies of sequences/genes in an attempt of genome restructuration and adaptation undergo several modifications resulting in karyotype and phenotypic changes (Parisod et al. 2009, Soltis and Soltis 2009, Lipman et al. 2013). In *Lathyrus nervosus* Lam. (Fabaceae), for example, it was observed karyotypic variations in different populations although the individuals showed the same chromosome number (Chalup et al. 2012).

The cytomolecular data here observed is the first relate for the genus. The number of 5S rDNA probes observed was in according to the expected number. Nevertheless, two additional marks of 45S rDNA was detected (6 instead of 4) reinforcing the hypothesis that chromosome structural rearrangements such as duplication, translocations and transpositions events might occurred after the chromosome doubling, increasing the number of 45S rDNA sites. Similar result was reported for *Byblis rorida* Lowrie & Conran (2n = 16) cytotypes (Fukushima et al. 2011). The observation of some individuals with additional sites of rDNA, suggested an increase in the number of the locus through structural rearrangements, since it was not observed any difference in chromosome number among the individuals investigated (Fukushima et al. 2011).

Alternatively, additional chromosomes (from unbalanced gamete) may also explain the number of 45S rDNA sites here observed for *Plectranthus barbatus*. Several authors have been discussed the stability of 45S and 5S rDNA (numbers, size and position) in the cytotypes formation. Reis et al. (2014), for example, described a polyploid complex for *Lippia alba* (Verbenaceae) and suggested that 45S rDNA sites are very variable (in general, the expected number of sites was not observed in polyploids due to deletions). On the other hand, taking the monoploid number of sites as reference, the 5S rDNA was more stable following the expected number according to the ploidy level. In the common bean *Phaseolus vulgaris* L. (Fabaceae), Pedrosa-Harand et al. (2006) observed that the number of 45S rDNA sites varied from 6 to 18 per accession. According to the authors, amplifications and deletions would be the probable reason of the wide variation observed. Regarding to the size of rDNA sites, some differences may be due to the partial amplification and deletion of some sites, already described as a common phenomenon in ribosomal sites of plant chromosomes (Roa and Guerra 2012).

Flow cytometry analysis indicated that the *Plectranthus barbatus* genome size is relatively small comparing with other Lamiaceae species. Taking all estimations described so far from 25 genera, the 1C value ranged from 0.28 to 6.24 pg. (Bennett 1972, Galbraith et al. 1983, Olszewska and Osiecka 1983, Ohri and Kumar 1986, Suda et al. 2003, Ohri et al. 2004, Rosenbaumová et al. 2004, Suda et al. 2005, Schmidt-Lebuhn et al. 2008, Kubesoval et al. 2010, Mahdavi and Karimzadeh 2010, Siljak-Yakovlev et al. 2010, Temsch et al. 2010, Bainard et al. 2011, Vesely et al. 2011). The present study reported is the first DNA content estimation for *Plectranthus barbatus* and also the first estimation for the genus *Plectranthus*.

Although there are several estimations of plant genome sizes, few of them reported the AT/GC genome composition, being the most detailed studies performed by Meister and Martin (2007) and Smarda and Bures (2012). Recently the average of GC composition for different kingdoms was reported begin possible to observe that monocots showed 45.15% of GC while dicots showed 34.36% of the same bases (Li and Du 2014). In *Plectranthus barbatus*, GC content estimation was quite close to the most of dicots (Carels 2005). It was also possible to observe that part of the GC base composition of *Plectranthus barbatus* genome (1.18%) corresponds to the bright blocks of CMA_3 _constitutive heterochromatin. This is also the first report of chromosome banding data for the genus.

In addition to understand the biology and the evolution of plant species, the characterization of chromosome number and DNA content can be very interesting, especially when different cytotypes had been described for a medicinal species. Studying different accessions of *Lippia alba* (Mill.) N. E. Brown (Verbenaceae), an important medicinal plant in Brazil, it was observed that different chemotypes, but morphologically similar, showed different major component of the essential oil (Viccini et al. 2014). While diploids and tetraploids possess citral as the major component, triploids cytotypes had linalool. Considering that linalool and citral have different medicinal applications, the characterization of these plants is very important, helping the correct medicinal use of the species (Viccini et al. 2014, Reis et al. 2014).

In addition to the new data here described more species of the genus *Plecthrantus* and of the Lamiaceae family should be investigated once no detailed cytogenetic data is available. The increase in the number of taxa will be very important for a better understanding of the biology and the evolutionary relationship within this important medicinal plant group. Other cytotypes and possible chemotypes of *Plectranthus barbatus* are up to know under-characterized.
